# Novel kinase fusion transcripts found in endometrial cancer

**DOI:** 10.1038/srep18657

**Published:** 2015-12-22

**Authors:** Ryo Tamura, Kosuke Yoshihara, Kaoru Yamawaki, Kazuaki Suda, Tatsuya Ishiguro, Sosuke Adachi, Shujiro Okuda, Ituro Inoue, Roel G. W. Verhaak, Takayuki Enomoto

**Affiliations:** 1 Department of Obstetrics and Gynecology, Niigata University Graduate School of Medical and Dental Sciences, Niigata, Japan; 2 Department of Bioinformatics, Niigata University Graduate School of Medical and Dental Sciences, Niigata, Japan; 3 Division of Human Genetics, National Institute of Genetics, Mishima, Japan; 4 Department of Bioinformatics and Computational Biology, The University of Texas MD Anderson Cancer Center, Houston, TX, USA; 5 Department of Genome Medicine, The University of Texas MD Anderson Cancer Center, Houston, TX, USA

## Abstract

Recent advances in RNA-sequencing technology have enabled the discovery of gene fusion transcripts in the transcriptome of cancer cells. However, it remains difficult to differentiate the therapeutically targetable fusions from passenger events. We have analyzed RNA-sequencing data and DNA copy number data from 25 endometrial cancer cell lines to identify potential therapeutically targetable fusion transcripts, and have identified 124 high-confidence fusion transcripts, of which 69% are associated with gene amplifications. As targetable fusion candidates, we focused on three in-frame kinase fusion transcripts that retain a kinase domain (*CPQ-PRKDC*, *CAPZA2*-*MET*, and *VGLL4*-*PRKG1*). We detected only *CPQ-PRKDC* fusion transcript in three of 122 primary endometrial cancer tissues. Cell proliferation of the fusion-positive cell line was inhibited by knocking down the expression of wild-type *PRKDC* but not by blocking the *CPQ*-*PRKDC* fusion transcript expression. Quantitative real-time RT-PCR demonstrated that the expression of the *CPQ*-*PRKDC* fusion transcript was significantly lower than that of wild-type *PRKDC*, corresponding to a low transcript allele fraction of this fusion, based on RNA-sequencing read counts. In endometrial cancers, the *CPQ-PRKDC* fusion transcript may be a passenger aberration related to gene amplification. Our findings suggest that transcript allele fraction is a useful predictor to find bona-fide therapeutic-targetable fusion transcripts.

Fusion genes resulting from chromosomal rearrangements have an important role in the genesis of some types of tumors and have been recognized as important diagnostic and prognostic parameters in various types of malignant tumors^1^,^2^. Fusion genes that are composed of protein kinase genes, such as anaplastic lymphoma receptor tyrosine kinase (*ALK*), ROS proto-oncogene 1, receptor tyrosine kinase (*ROS1*), ret proto-oncogene (*RET*), and fibroblast growth factor receptor (*FGFR*), have attracted significant attention as therapeutic drug targets^3^,^4^,^5^,^6^. Indeed, the echinoderm microtubule-associated protein-like 4 (*EML4*)-*ALK* gene fusion has been detected in a small subset of non-small cell lung cancers^7^, and the efficacy of using ALK inhibitors to treat lung cancer patients who harbor the *EML4-ALK* fusion has been evident in clinical trials^8^,^9^.

Recent advancements in sequencing technology have enabled comprehensive identification of fusion genes in human cancers, and candidate therapeutic targets have now been reported across many types of tumors^10^,^11^. However, the functional evaluation of these fusion genes detected by whole-genome or transcriptome sequencing has been largely insufficient, if attempted at all, and it thus remains difficult to differentiate bona fide therapeutically targetable fusions from among the many candidates.

Endometrial cancer is currently the fourth most common cancer in women, and its incidence is rapidly increasing in Japan (http://ganjoho.jp/en/index.html). Although many cases of endometrial cancer are detectable at an early stage and show favorable outcomes, some are resistant to chemotherapy or radiotherapy, leading to a poor prognosis. To investigate the basis of this heterogeneity in endometrial cancer outcomes, several groups have performed genomic or transcriptomic analyses^12^,^13^,^14^,^15^,^16^. For example, The Cancer Genome Atlas (TCGA) Research Network divides endometrial cancers into four categories, based on the integrated analysis of genomic, transcriptomic and proteomic data: polymerase ε (*POLE*) ultramutated, microsatellite-instability hypermutated, copy-number low, and copy-number high^12^. Despite this extensive molecular exploration of endometrial cancer, there is at present no effective therapeutic molecular target for this disease.

We have combined RNA-sequencing data from 25 endometrial cancer cell lines with array data of matched small nucleotide polymorphisms (SNPs) obtained from the Cancer Cell Line Encyclopedia (CCLE, http://www.broadinstitute.org/ccle) to define the landscape of gene-fusion transcripts in endometrial cancer cells, with the intention of discovering new potential therapeutic molecular targets for endometrial cancer.

## Results

### Detection of fusion transcripts

To identify high-confidence fusion transcripts, we applied the Pipeline for RNA sequencing Data Analysis (PRADA, http://bioinformatics.mdanderson.org/Software/PRADA/)^17^ software program to the CCLE RNA-sequencing data for 25 endometrial cancer cell lines. The detail of RNA sequencing data including such as data quality and origin of cell line was shown in Supplementary Table S1. We detected 287 fusion transcripts supported by at least two discordant read pairs plus one perfectly matched junction-spanning read, with the other end of the read pair mapping to either of the fusion gene partners. To reduce the number of false positive predictions, we filtered the fusion transcripts according to partner gene homology. We used BLASTn (Basic Local Alignment Search Tool-Nucleotides) to determine the homology between partnered genes, and removed 151 hits consisting of gene pairs with high similarity. After removing the overlapping fusion pairs, with 158 kinds of fusion transcripts discovered in 364 normal tissues samples^11^, we identified 124 cancer-specific fusion transcripts from the 25 endometrial cancer cell lines (Supplementary Table S2).

### Landscape of fusion transcripts in 25 endometrial cancer cell lines

**Figure 1:**
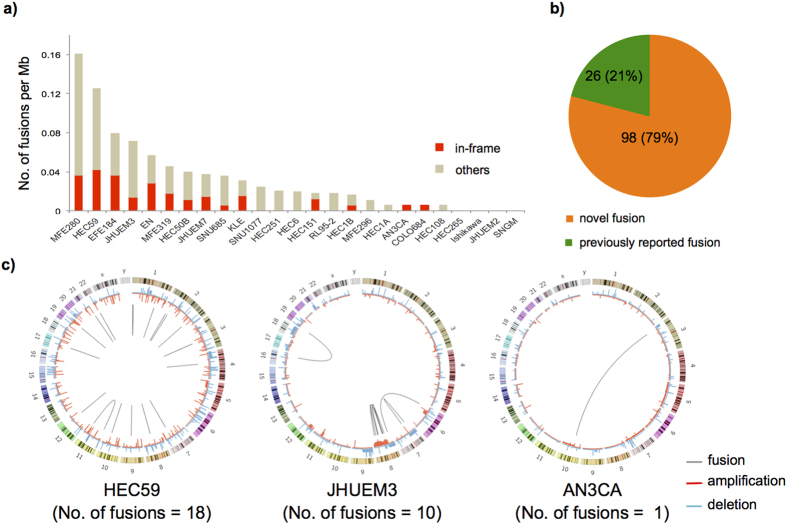
An overview of fusion transcripts found in endometrial cancer cell lines. (**a**) Histogram of the number of fusion transcripts per Mb in 25 endometrial cancer cell lines. (**b**) The percentage of novel fusion transcripts in endometrial cancer cell lines. Previously reported fusions were defined on the basis of TCGA Fusion Gene Data Portal (http://www.tumorfusions.org). (**c**) Circos plots indicate the landscape of fusion transcripts in JHUEM3, HEC59, and AN3CA cell lines. The outermost circle represents chromosomes and cytogenetic bands. The next circle represents copy number alterations identified using GISTIC. Green indicates deletion, and red indicates amplification. Each connection with gray lines in an innermost circle indicates fusion transcripts.

The frequency of fusion transcripts per each cell line is shown in Fig. 1a. Four cell lines did not harbor any detectable, expressed fusion transcripts, and 14 cell lines had at least one in-frame fusion transcript that could result in a functional protein. As of May 1, 2015, only 26 of 124 cancer-specific fusion transcripts were previously reported in the TCGA Fusion Gene Data Portal (http://www.tumorfusions.org) (Fig. 1b and Supplementary Table S2).

To visualize the fusion-gene profile for each cell line, we used circos plots^18^ to represent the fusion events. Based on Genomic Identification of Significant Targets in Cancer 2.0 (GISTIC2)^19^ analysis, we also evaluated alterations in the fusion gene copy numbers (Fig. 1c and Supplementary Fig. S1). Overall, intra-chromosomal gene fusions were predominant in the endometrial cancer cell lines. Chromosome 8q harbored 70% of the fusion transcripts in the JHUEM3 endometrial cancer cell line corresponding to broad 8q regional amplification, suggesting the occurrence of chromothripsis^20^.

**Figure 2:**
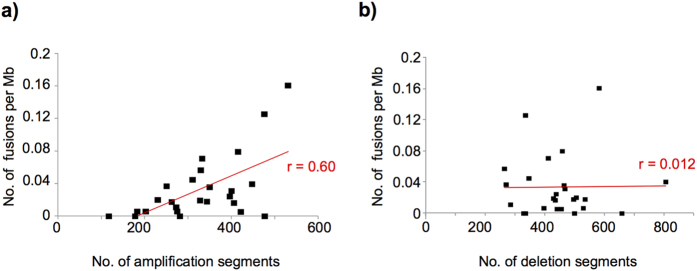
Association of copy number alterations with fusions. Correlation between the number of amplified (**a**) or deleted (**b**) segments and fusion transcripts per Mb in 25 endometrial cancer cell lines.

Next, we investigated the association between copy number alterations and fusion transcripts. We found a significant positive correlation between the number of fusion transcripts and the number of amplification segments (Fig. 2a), but not deletion segments (Fig. 2b). At least one of the fused genes was amplified in the cell line in 76 (69%) of the 109 fusions pairs; whereas deletion of one of the fused genes was detected in only 23 (20%) of the 109 fusion pairs. Although the frequency of in-frame or inter-chromosomal fusions was lower than that of out-of-frame or intra-chromosomal fusions, there was no significant association between these fusion types and copy number alterations (Supplementary Fig. S2).

**Figure 3:**
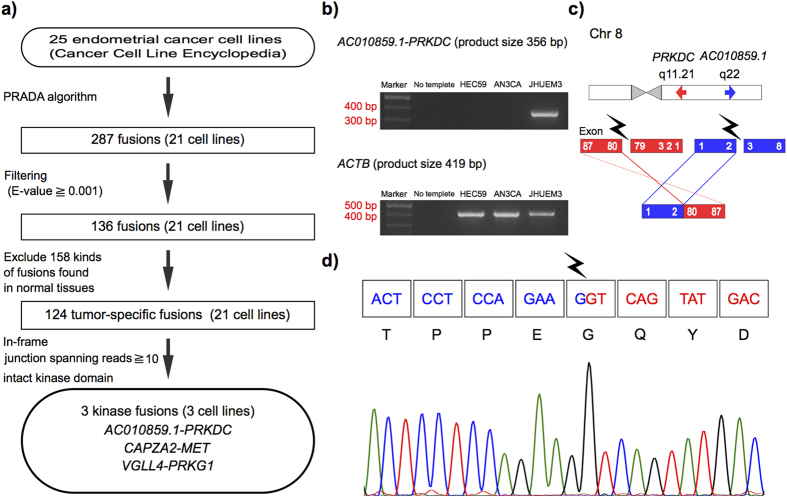
Validation of targetable fusions. (**a**) Flowchart for discovering targetable fusion transcripts. (**b**) Identification of *CPQ-PRKDC* fusion transcripts in JHUEM3 using RT-PCR. Marker represents a 100 bp DNA ladder. *ACTB* was used as the internal control for RT-PCR. (**c**) Schematics of the *CPQ-PRKDC* fusion transcripts. Regions corresponding to *CPQ* or *PRKDC* are shown in red or blue, respectively. (**d**) Sanger sequencing chromatogram showing the reading frame at the breakpoint and putative translation of the fusion protein in the fusion-positive cell line.

To focus on therapeutically targetable fusion transcripts in endometrial cancer, from the initial list of 124 fusion transcripts, we identified three in-frame kinase fusion transcripts (*CPQ*-*PRKDC*, *VGLL4*-*PRKG1*, and *CAPZA2*-*MET*) that were supported by more than 10 junction-spanning reads, which included an intact kinase domain (Fig. 3a)^21^. The *CPQ-PRKDC* fusion transcript has not been reported previously.

### Validation of fusion transcripts

To evaluate the authenticity and validity of our list of gene fusion transcripts, we used three human endometrial cancer cell lines (AN3CA, HEC59 and JHUEM3) that had in-frame kinase fusions. We performed Sanger sequencing of RT-PCR amplification products that spanned fusion junction points to validate all transcripts detected in the three cell lines. Although the RT step has possibility to produce artifactual fusion transcripts, we detected 27 (93.1%) of the 29 fusion transcripts by using Sanger sequencing RT-PCR amplification products (Fig. 3b–d). In particular, all fusion transcripts showing more than 10 junction-spanning reads^21^ were identifiable.

To assess the frequency of occurrence of the three in-frame kinase fusions (*CPQ*-*PRKDC*, *VGLL4*-*PRKG1*, *CAPZA2*-*MET*) in clinical samples, we conducted RT-PCR and Sanger sequencing for 122 primary endometrial cancer tissues. We detected *CPQ*-*PRKDC* fusion transcripts in three (2.5%) of the 122 primary tumor tissues; whereas we failed to detect *VGLL4*-*PRKG1* or *CAPZA2*-*MET* fusion transcripts in any of the clinical samples (Table 1). To rule out congenitally inherited gene fusions, we next conducted RT-PCR using paired normal tissues obtained from patients whose endometrial cancer tissues harbored the *CPQ*-*PRKDC* fusion transcript, and confirmed that this fusion event was somatic and tumor-specific (Supplementary Fig. S3).

### Characterization of the novel *CPQ*-*PRKDC* fusion transcript

We used microarray data for mRNA expression, downloaded from the CCLE data repository, to examine the expression of *PRKDC* mRNA in endometrial cancer cell lines. The expression of the total *PRKDC* transcript in the *CPQ*-*PRKDC* fusion-positive cell line was relatively higher than that in the other endometrial cancer cell lines that did not have the *CPQ*-*PRKDC* fusion (Supplementary Fig. S4a). In addition, we found amplification of *PRKDC* gene in the *CPQ-PRKDC* fusion-positive cell line (Supplementary Fig. S4b). Next we examined the frequency of *PRKDC* gene alterations in TCGA endometrial cancer samples using cBioPortal^22^ (http://www.cbioportal.org). Of 240 TCGA endometrial cancer samples, three samples (1.3%) showed *PRKDC* amplifications with concurrent overexpression. Although 24 samples (10.0%) harbored *PRKDC* somatic mutations, there was no hotspots in *PRKDC* mutations (Supplementary Fig. S4c).

**Figure 4:**
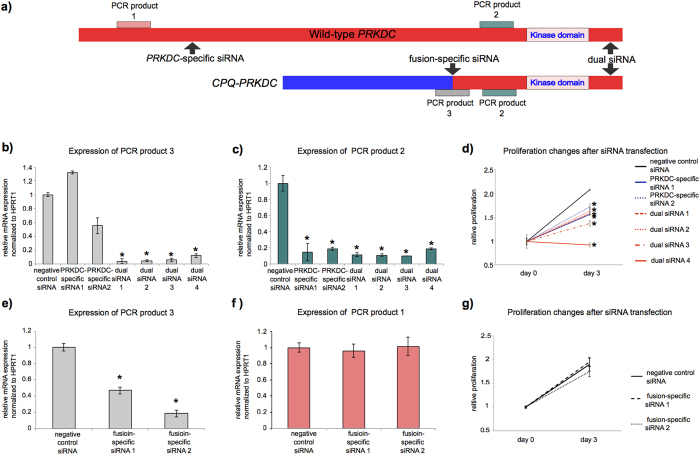
Knockdown of *PRKDC* and *CPQ-PRKDC* by siRNA. (**a**) Schematics of *CPQ-PRKDC* fusion transcripts and wild-type *PRKDC*. PRKDC-specific, fusion-specific and dual siRNAs were used in the knockdown experiments. Expression levels of the wild-type *PRKDC* (PCR product 1, beige), dual *PRKDC* (PCR product 2, green) and *CPQ-PRKDC* (PCR product 3, pink) were measured using appropriate primers. (**b**,**c**) *PRKDC* siRNAs were transfected to JHUEM3 harboring *CPQ-PRKDC* fusion transcript. The mRNA expressions of *CPQ-PRKDC* and dual *PRKDC* were assessed by quantitative real-time RT-PCR. Data represent the mean and error bar of triplicate independent experiments (asterisk denotes *p* < 0.01). (**d**) The effect of siRNA-mediated down-regulation of *PRKDC* on JHUEM3 cell proliferation. The cell proliferation was assessed by CellTiter-Glo assay. Data represent the mean and error bar of quadruplicate independent experiments (asterisk denotes *p* < 0.01). (**e**,**f**) Fusion-specific siRNA were transfected into JHUEM3 harboring an *CPQ-PRKDC* fusion. The mRNA expression levels of *CPQ-PRKDC* and wild-type *PRKDC* were assessed by quantitative real-time RT-PCR. Data represent the mean and error bar of triplicate independent experiments (asterisk denotes *p* < 0.01). (**g**) The effect of siRNA-mediated down-regulation of *CPQ-PRKDC* on JHUEM3 cell proliferation.

We investigated an association between *PRKDC* and *CPQ-PRKDC* expression and cell proliferation. To knock down the expression of *PRKDC* and *CPQ*-*PRKDC*, we prepared three kinds of small interfering RNA (siRNA), as follows: *PRKDC*-specific siRNA, dual siRNA and fusion-specific siRNA (Fig. 4a). Quantitative RT-PCR revealed that *PRKDC*-specific siRNA suppressed *PRKDC* expression but not *CPQ-PRKDC* fusion transcript expression, and that dual siRNA knockdown blocked the expression of both the *PRKDC* and *CPQ*-*PRKDC* transcripts (Fig. 4b,c). Suppression of *PRKDC* expression by *PRKDC*-specific siRNA or by dual siRNA inhibited cell proliferation (Fig. 4d). In addition, after transfection with dual siRNA, caspase 3/7 activity was noticeably higher, compared to the negative siRNA control (Supplementary Fig. S5a), suggesting that a DNA repair defect caused by loss of *PRKDC* expression might be leading to increased apoptosis.

When we transfected fusion-specific siRNA into the cell line expressing the *CPQ*-*PRKDC* fusion transcript, *CPQ-PRKDC* expression was suppressed, but not the wild-type *PRKDC* expression (Fig. 4e,f). However, knockdown of *CPQ*-*PRKDC* fusion transcript expression neither inhibited cell proliferation nor elevated caspase 3/7 activity in the fusion-positive cell line (Fig. 4g, Supplementary Fig. S5b).

In the cell line harboring the *CPQ*-*PRKDC* fusion transcript, the expression of the fusion transcript was definitely lower than that of the wild-type *PRKDC* transcript. Similarly, the two endometrial cancer tissues expressing the *CPQ*-*PRKDC* fusion transcript also showed relatively lower expression of the *CPQ*-*PRKDC* fusion transcript (Supplementary Table S3). Corresponding to the mRNA expression level of fusion transcripts, Western blot did not detect protein expression of the three fusion transcripts (Supplementary Fig. S6).

### Differences in transcript allele fraction between endometrial cancer cell lines and clinical samples

**Figure 5:**
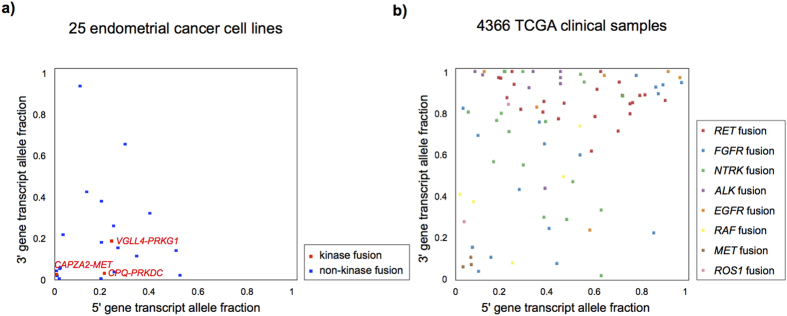
Comparison of transcript allele fraction (TAF) between endometrial cancer cell lines and TCGA clinical samples. (**a**) TAF value for each fusion transcript in endometrial cancer cell lines. Each plot represents the TAF of fusion transcripts that were supported by more than 10 junction-spanning reads. X- and y-axes represent TAF of 5′ and 3′ genes. Blue plots and gray squares represent in-frame and out-of-frame fusion transcripts, respectively. The three in-frame kinase fusion transcripts are highlighted in red. (**b**) TAF for 82 recurrent in-frame kinase fusion transcripts in TCGA clinical samples that were supported by more than 10 junction-spanning reads and that retained the kinase domain.

To compare the transcript allele fraction (TAF) of the kinase in-frame fusion transcripts between endometrial cancer cell lines and TCGA clinical samples, we used 7,887 fusion transcripts detected in 4,366 TCGA samples across 13 tumor types^11^. We extracted 82 recurrent in-frame kinase fusions that were supported by more than ten junction-spanning reads and retained an intact kinase domain. Compared to the TAF scores of the recurrent in-frame kinase fusion transcripts, including therapeutically targetable fusion transcripts (Supplementary Table S4). Despite high tumor purity based on DNA copy number alterations^23^, the scores of the three kinase in-frame fusions in endometrial cancer cell lines were lower (Fig. 5).

## Discussion

Our study of 25 human endometrial cancer cell lines through paired-end RNA-sequencing data analysis has provided a list of high-confidence fusion transcripts and displayed the diversity of fusion transcripts and the association of fusion transcripts with gene amplification events.

TCGA Research Network performed an integrated analysis of genomic, transcriptomic and proteomic data obtained from 373 endometrial cancers to gain a better understanding of the molecular characteristics of this type of tumor^12^. Although 49 fusion transcripts, including *BCL*-related fusions, were identified in 106 endometrial cancers by using low-pass paired-end whole-genome sequencing data, there were no fusion transcripts that overlapped between the 106 TCGA clinical samples of endometrial cancer and the 25 endometrial cancer cell lines. There was no significant difference in RNA sequencing data quality between CCLE and TCGA (data not shown). This discrepancy might reflect differences in the types of sequencing data (low-pass paired-end whole genome data versus paired-end RNA-sequencing data) and the samples (clinically resected tissue versus cell line). Indeed, two of the three in-frame kinase fusions in the endometrial cancer cell lines were not detected in any of the 122 clinical samples. In this regard, Domcke *et al.*^24^ reported striking differences in the molecular characteristics between commonly used ovarian cancer cell lines and primary high-grade serous ovarian cancer samples. Genomic alterations in culture-adapted cancer cell lines might not reflect the genomic alterations present in clinically resected samples. Another reason of the discrepancy between cell lines and clinical samples might be the small statistical power to discover new fusions in the small sample set. However, a list of fusion transcripts in cancer cell lines might still be a useful resource for the functional analysis of fusions detected across many tumor types^21^,^25^.

Gene fusions involving kinase genes are receiving abundant attention as potential therapeutic molecular targets. Although the frequency of recurrent kinase fusion transcripts is generally lower than that of somatic mutations in malignant solid tumors, specific kinase inhibitors have been shown to have a curative effect on tumors that harbor related kinase gene fusions. For example, the development of imatinib mesylate, the first tyrosine kinase inhibitor targeting the BCR-ABL1 fusion protein, has dramatically improved prognosis in patients with chronic myeloid leukemia^26^. Today, non-small cell lung cancer patients with an *EML4*-*ALK* fusion are routinely treated with ALK inhibitors as first-line chemotherapy^8^,^9^, even though this fusion was first reported only eight years ago. These success stories have accelerated our efforts to discover novel therapeutically targetable kinase fusions by using whole-genome or transcriptome data analysis.

We found that intra-chromosomal gene fusions predominated in the endometrial cancer cell lines, which is similar to what has been found in other cancers^10^. Chromosome 8q harbored 70% of the fusion transcripts in the JHUEM3 endometrial cancer cell line, corresponding to broad 8q amplification in that cell line. This suggests the occurrence of chromothripsis, a phenomenon by which up to thousands of clustered chromosomal rearrangements occur in a single event in localized genomic regions in one chromosome. Chromothripsis is known to be involved in both cancer and congenital diseases^19^.

Of particular interest to us was the novel kinase fusion *CPQ-PRKDC*, which we also detected in 2.5% of primary endometrial cancer samples. The DNA-dependent protein kinase catalytic subunit (DNA-PKcs) encoded by *PRKDC* is a critical component of the DNA repair machinery and thus plays an important role in maintaining genome integrity^27^,^28^. DNA-PK is also associated with AKT phosphorylation, leading to the activation of the AKT signaling pathway^29^,^30^. In addition, recent reports have demonstrated synthetic lethal interaction between *PRKDC* and some genes such as *ATM*^31^, *MYC*^32^, or *MSH3*^33^, suggesting the significance of *PRKDC* as a druggable target. Although the *CPQ-PRKDC* fusion is an attractive candidate for a therapeutic target in endometrial cancer, our knock-down experiments and Western blot analysis demonstrated that inhibiting *CPQ-PRKDC*- positive cell line proliferation was caused by suppression of the wild-type *PRKDC* expression and not suppression of the fusion transcript, which suggests that this fusion transcript might be a passenger alteration.

The TAF provides a quantitative assessment of the relative expression levels of the wild-type and fusion transcripts. The TAF of *PRKDC* was lower than that of other clinically applicable kinase fusions, such as *ALK*^8^,^9^*, RET *34,35*, FGFR*^36^,^37^, and *ROS1*^38^ (Fig. 5b). When we calculate the TAF score using RNA-sequencing data from clinical tumor samples, we should consider the tumor purity of clinical samples because mRNA expression derived from non-tumor cells would lead to the underestimation of the TAF of a tumor-specific fusion transcript^39^. A previous report has demonstrated the association between fusion transcripts and genomic alterations in breast cancer^40^. Kalyana-Sundaram *et al.*^41^ have also reported that amplicon-associated *EGFR* or *RPS6KB1* fusion transcripts without a kinase domain represented low TAF values and were passenger aberrations in breast cancer cell lines. Corresponding to the previous work, out study demonstrated that HEC59 harboring *CAPZA2*-*MET* fusion transcript had high amplification of *MET*, suggesting this fusion was passenger aberration. The information of kinase domain in fusion transcript should also be considered to detect therapeutically targetable fusion genes and Stransky *et al.* have succeeded to discover recurrent *MET* fusions with a putative activating structure in the TCGA dataset^10^.

We have described the landscape of fusion transcripts in endometrial cancer by using RNA-sequencing data. Unfortunately, we could not identify therapeutically targetable kinase fusions in a small set of 25 endometrial cancer cell lines. To distinguish fusions that drive tumorigenesis from the more numerous incidental passenger fusions, researchers should weigh the TAF, gene copy number alterations, and retention of functional domains. Our findings suggest that the TAF is a good predictor for determining bona fide targetable fusion transcripts when applying fusion detection methods that use RNA-sequencing data.

## Methods

### Identification of fusion transcripts

We downloaded the RNA-sequencing data for 25 endometrial cancer cell lines from the CCLE^42^ at the Cancer Genomics Hub (https://cghub.ucsc.edu)^43^. We used PRADA as previously reported^11^ to detect fusion transcripts in the database. We extracted fusion transcripts characterized by (1) at least two discordant read pairs, (2) at least one junction-spanning read, and (3) no high gene homology between each fusion gene partner (E-value > 0.001). Next, we extracted tumor-specific fusion transcripts by removing the fusion transcripts that overlapped with 158 fusion transcripts discovered in 364 normal tissue samples.

We also downloaded the copy number data of endometrial cancer cell lines from CCLE. We integrated the fusion transcript data and copy number data to investigate the association between fused genes and copy number alterations.

### Samples

The three human cancer cell lines (HEC59, JHUM3, and AN3CA) were purchased from the Japanese Collection of Research Bioresources Cell Bank, RIKEN Cell Bank, and American Type Culture Collection, respectively. All cell lines were cultured in Dulbecco’s modified Eagle medium, supplemented with 10% fetal bovine serum, 50 IU/mL penicillin, and 50 μg/mL streptomycin.

Tissues from 122 Japanese patients who were diagnosed with endometrial cancer between 2006 and 2014 at Niigata University Medical and Dental Hospital were included in this study. Fresh frozen samples were obtained from primary tumor tissues during surgery prior to chemotherapy. The histologic characteristics of surgically resected specimens were assessed on formalin-fixed and paraffin-embedded hematoxylin and eosin sections by a gynecologic pathologist. A total of 122 samples (109 endometrioid adenocarcinomas, 5 mixed adenocarcinomas, 4 carcinosarcomas, 2 undifferentiated adenocarcinomas, 1 serous adenocarcinoma and 1 unclassified adenocarcinoma of the uterine corpus) were analyzed. The institutional ethics review board at Niigata University approved this study. All patients provided written informed consent for the collection of samples and subsequent analysis. All experiments were performed in accordance with the approved guidelines.

### RT-PCR and Sanger sequencing

Total RNA was extracted from fresh frozen samples using TRIzol (Invitrogen, Carlsbad, CA, USA) and from cell lines using the RNeasy mini kit (Qiagen, Tokyo, Japan). Total RNA (1 μg) was reverse-transcribed into cDNA using Prime Script II Reverse Transcriptase (Takara Bio, Shiga, Japan). cDNA (corresponding to 10 ng total RNA) was subjected to PCR amplification using KAPA Taq DNA polymerase (KAPA Biosystems, Woburn, MA, USA) or Prime Star HS premix (Takara Bio). The reactions were carried out in a thermal cycler under the following conditions: 40 cycles at 95 °C for 30 sec, at 60 °C for 30 sec, and at 72 °C for 1 min, with a final extension at 72 °C for 1 min (KAPA Taq DNA polymerase). Alternatively, PCR was done for 40 cycles at 98 °C for 15 sec, at 68 °C for 1 min, and a final extension at 72 °C for 5 min (Prime Star HS premix).

Beta-actin (*ACTB*) was amplified to evaluate the efficiency of cDNA synthesis. PCR products were extracted and purified by NucleoSpin Gel and PCR Clean-up (Takara Bio) and were sequenced on an ABI 3130xl DNA Sequencer (Applied Biosystems, Foster City, CA, USA) using a BigDye

Terminator kit (Applied Biosystems). The PCR primers used in this study (shown in Supplementary Table S5) were designed using Primer3Plus (http://www.bioinformatics.nl/cgi-bin/primer3plus/primer3plus.cgi/).

### siRNA knock-down experiments

The negative control siRNA (with scrambled sequence, SI1022076)*, PRKDC*-specific siRNA (SI2663633, SI2224229), and dual siRNA (SI2224236, SI4436698, SI4436705, SI4436684) were purchased from Qiagen (Tokyo, Japan). Fusion-specific siRNA was designed using siDirect (http://sidirect2.rnai.jp). The siRNA sequences are shown in Supplementary Table S6. Quantitative real-time RT-PCR was performed using total RNA that was extracted after 72 hours of siRNA transfection. Cell proliferation and apoptosis were measured after 24 and 96 hours of siRNA transfection using the CellTiter Glo assay and the Caspase-Glo 3/7 assay, respectively (Promega, Mannheim, Germany) according to the manufacturer’s protocols.

### Quantitative real-time RT-PCR

Quantitative real-time RT-PCR was performed with a Thermal Cycler Dice Real-Time System TP800 2.01C (Takara Bio). cDNA (corresponding to 5 ng of total RNA) was subjected to real-time PCR amplification analysis using SYBR Green PCR mix (Applied Biosystems). Gene expression was tested in a set of 15 human housekeeping genes (Takara Bio) and *HPRT1* was selected as having the lowest variation in gene expression. The relative quantification method^44^ was used to measure the amounts of the respective genes normalized to *HPRT1*. All primers used in quantitative real-time RT-PCR are shown in Supplementary Table S7.

### Western blotting

Cells were lysed in lysis buffer (50 mM Tris at pH 8.0, 150 mM NaCl, 1% Nonidet P-40, 0.5% sodium deoxycholate, 0.1% SDS and 1 mM EDTA) supplemented with protease inhibitors. The lysates were electrophoresed using a 5–20% real gel plate (Bio Craft, Tokyo, Japan) and transferred to Immobilon-P membranes (Millipore, Darmstadt, Germany). Western blot analyses were performed with anti-*PRKDC* (#4602, Cell Signaling Technology, Tokyo, Japan), anti-*MET* (#3019, Cell Signaling Technology), anti-*PRKG1* (#5592, Cell Signaling Technology), and anti-*GAPDH* (MAB374, Millipore) antibodies.

### Statistical analysis

Data were expressed as the mean ± SD. Fisher’s exact test and Student t-test were used for evaluation of significance between data groups. P values less than 0.05 indicated statistical significance.

## Additional Information

**How to cite this article**: Tamura, R. *et al.* Novel kinase fusion transcripts found in endometrial cancer. *Sci. Rep.* **5**, 18657; doi: 10.1038/srep18657 (2015).

## Supplementary Material

Supplementary Information

Supplementary Table S1

Supplementary Table S2

Supplementary Table S4

## Figures and Tables

**Table 1:** Three kinase fusion transcripts in endometrial cancer.

Cell line	5′-Gene	3′-Gene	Locus 5′-Gene	Locus 3′-Gene	5′-Gene copy number*	3′-Gene copy number*	Discordant read (n)	Junction spanning read (n)	Frequency in clinical specimens
JHUEM3	*CPQ*	*PRKDC*	8q22.1	8q11.21	2	2	16	44	3/122 (2.5%)
HEC59	*CAPZA2*	*MET*	7q31.2	7q31.2	0	1	8	27	0/122 (0%)
AN3CA	*VGLL4*	*PRKG1*	3p25.3	10q21.1	−2	1	19	21	0/122 (0%)

^*^Putative copy number was determined by GISTIC2 (−2 = homozygous deletion; −1 = hemizygous deletion; 1 = low level gain; 2 = high level amplification)
