# Supplementary material for: Novel kinase fusion transcripts found in endometrial cancer
Source: Sci Rep. 2015 Dec 22;5:18657. doi: 10.1038/srep18657 (PMC4687039; doi:10.1038/srep18657)
Supplement: Supplementary Information [file srep18657-s1.doc]

**Supplementary Information**

**Novel kinase fusion transcripts found in endometrial cancer**

**Ryo Tamura^1^, Kosuke Yoshihara^1^, Kaoru Yamawaki^1^, Kazuaki Suda^1^, Tatsuya Ishiguro^1^,**

**Sosuke Adachi^1^, Shujiro Okuda^2^, Ituro Inoue^3^, Roel GW Verhaak^4, 5^, and Takayuki**

**Enomoto^1^**

**1. Department of Obstetrics and Gynecology, Niigata University Graduate School of Medical and Dental Sciences, Niigata, Japan**

**2. Department of Bioinformatics, Niigata University Graduate School of Medical and Dental Sciences, Niigata, Japan**

**3. Division of Human Genetics, National Institute of Genetics, Mishima, Japan**

**4. Department of Bioinformatics and Computational Biology, The University of Texas MD Anderson Cancer Center, Houston, TX, USA**

**5. Department of Genome Medicine, The University of Texas MD Anderson Cancer Center, Houston, TX, USA**

**Supplementary Figure S1**

**Circos plots representing genomic alterations in 18 cell lines harboring at least one fusion transcript**

Circos plots of 18 cell lines with at least one fusion transcript, except HEC59, JHUEM3 and AN3CA.


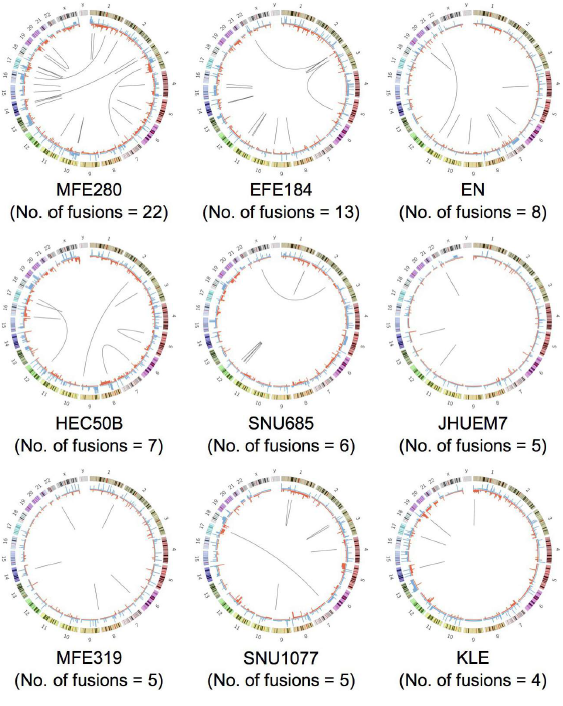


**
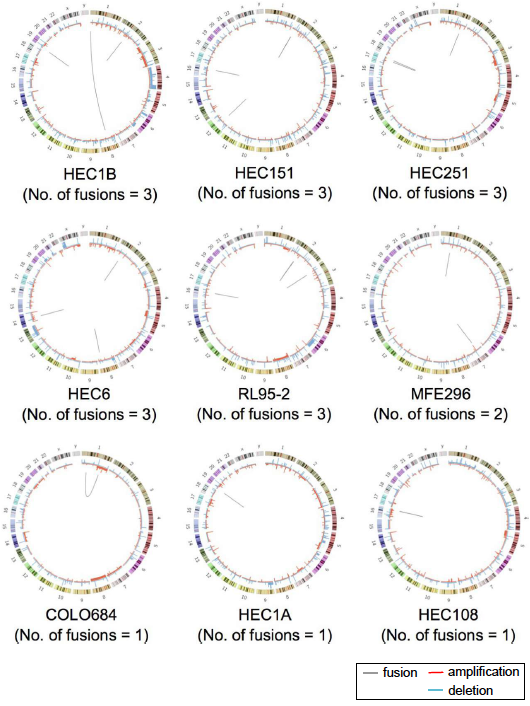
**

**Supplementary Figure S2**

**The details of copy number alterations in fused genes**

1. Pie chart shows the distribution of amplification status of fused genes in fusion transcripts. Copy number data were available for fused genes of 109 of the 124 fusion transcripts. Bar graph shows the association between in-frame or intra-chromosomal fusions and gene copy number alterations in endometrial cancer cell lines. The segments in orange and green show amplification and deletion, respectively, in at least one gene constituting the fusion transcript. The significance of differences was assessed by Fisher’s exact test.
2. Pie chart and bar graph show deletion status of fused genes in fusion transcript.

**Supplementary Figure S3**

**The expression of *CPQ-PRKDC* fusion transcripts in endometrial tumors and normal tissues**

Normal tissue was available in 5 of 7 cases with fusion-positive tumor samples. Five endometrial cancer samples harboring *CPQ-PRKDC* fusion transcripts (T) are shown, with 5 corresponding non-cancer tissues (N). Negative and positive controls are denoted as NTC and P, respectively.


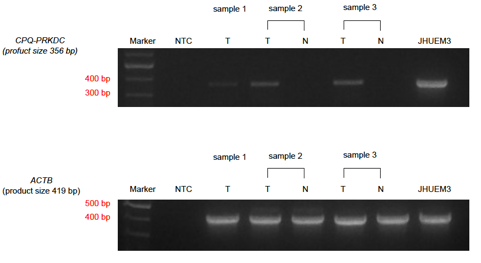


**Supplementary Figure S4**

**Molecular characterization of the *PRKDC* gene constituting the *CPQ-PRKDC* fusion transcript**

a) Expression of *PRKDC* mRNA in endometrial cancer cell lines

The fusion-positive cell line (JHUEM3) is highlighted in red. Data represent the z-score (indicating number of standard deviations away from the mean of expression in the reference genes) among endometrial cancer cell lines.

b) Copy number alteration of *PRKDC* in JHUEM3

Amplification is highlighted in red. Color intensity indicates the degree of gene amplification; red arrow represents the junction point of the *CPQ-PRKDC* fusion transcript genes.

c) Gene alterations of *PRKDC* in 240 clinical samples from TCGA
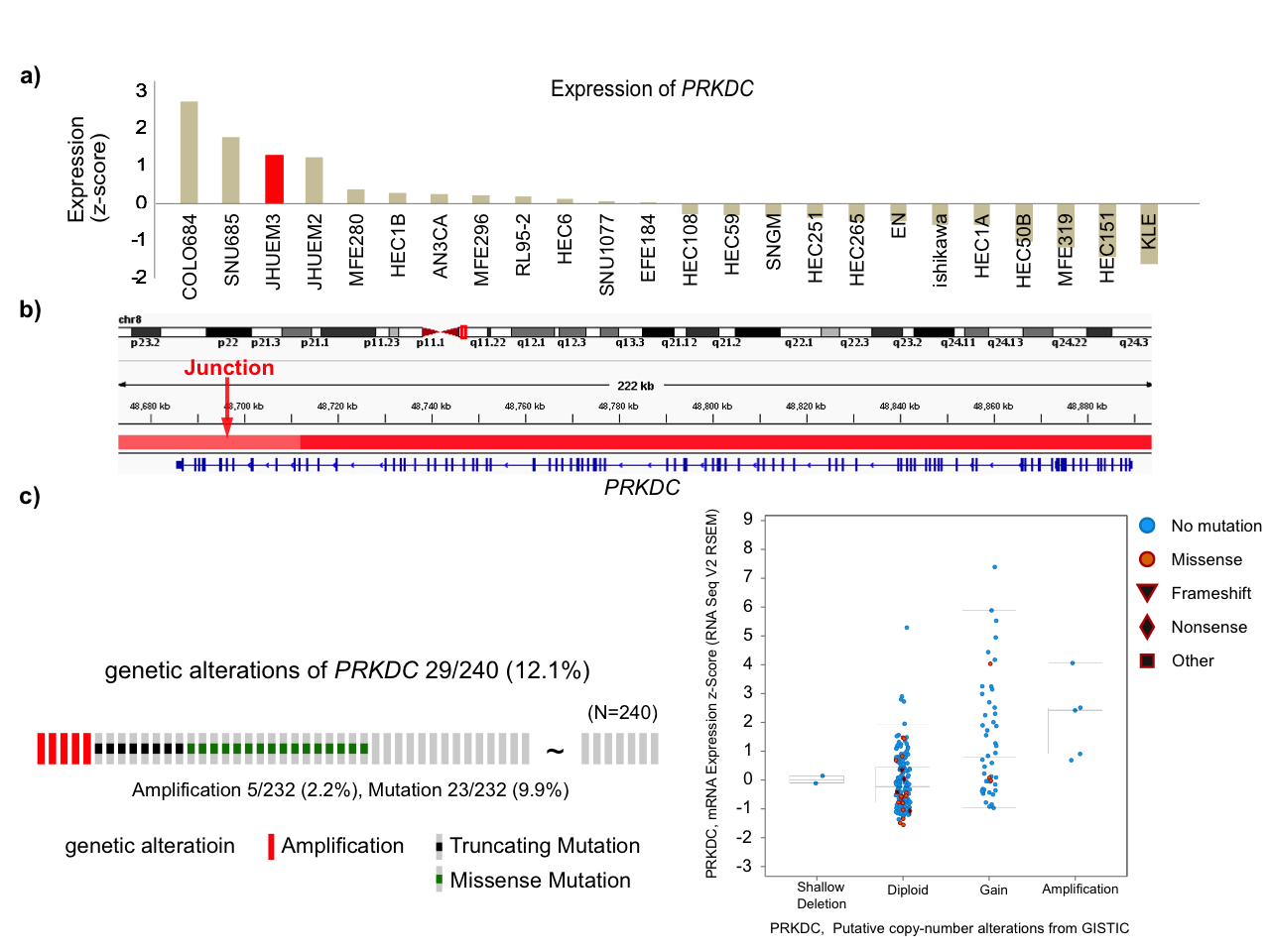


**Supplementary Figure S5**

**Caspase 3/7 activity after siRNA transfection**

Caspase-3/7 activation was significantly elevated in the *CPQ-PRKDC* fusion-positive cell line after transfection with dual siRNA, but not by fusion-specific siRNA.

**Supplementary Figure S6**

**No detection of fusion proteins**

Western blot showing the undetectable protein level of three kinase fusion genes (*CPQ-PRKDC*, *CAPZA2-MET*, and *VGLL4-PRKG1*).

**Supplementary Table S1**

**The detail of RNA sequencing data from 25 endometrial cancer cell lines**

**(Submitted as Supplementary Dataset)**

**Supplementary Table S2**

**All of fusion transcripts in endometrial cancer cell lines**

**(Submitted as Supplementary Dataset)**

**Supplementary Table S3**

**Relative expression of *CPQ-PRKDC* in JHUEM3 and clinical samples**

**Supplementary Table S4.**

**In-frame kinase fusions in 4336 TCGA clinical samples that supported more than 10 JSR and retained kinase domain**

**(Submitted as Supplementary Dataset)**

**Supplementary Table S5. Primer sequences for 27 validated fusion transcripts**

**Supplementary Table S6**

**siRNA target sequences**

**Supplementary Table S7**

**Primer sequences for quantitative real-time RT-PCR**
